# Supplementary material for: Chromothripsis during telomere crisis is independent of NHEJ, and consistent with a replicative origin
Source: Genome Res. 2019 May;29(5):737–49. doi: 10.1101/gr.240705.118 (PMC6499312; doi:10.1101/gr.240705.118)
Supplement: Supplemental Material [file supp_gr.240705.118_Supplemental_file_1.zip › contigs/annotated_contigs/DB78/contig.2.DB78_length_515_mean_cov_7.57281553398.docx]

**DB78_length_515_mean_cov_7.57281553398**

```
AAAACAAAGTTATTCCAAAAATGAGGACATCCTCCTGGCATCTGAGTCCTCACCCCATGTGCCACGGTGGCCGCTTCTGCCGTCCTCCA
 >chr7:5519719-5520066 - E=2e-197
CCTCCAGGCGCTCCTAGAGCTGTCCCTGGGCCAGTGGCTTCCAAAGGGGGCTGTAGTTGGGCCCTGCTAGCCTGGACCGCCGCCCTGGC

CGCTCTTGGTGAAGGGCCCCTTGTCCAGCCCGCCTTCCTCCTCCTGGGTTTCCGTGTGACAGATGCCCCGTCCTGTGGGGTGGTGTCTC

ACATTTGCTTTGCTGTTAAAAAATGGGGTACACCATCCCCAGGCCTCCAATCACCGGCCCTGCCCCTGAGTGGGGATGGT|A|CCTGTC
                                                                                  >chr7
CTACCCAACCAGACTGCCACTTACAAAGCAGGAGTCAACAACCAAAGGCTGCGGGCTGCTCCTTCTGGCTGCACAACCTCCCTCTCCCA
:5629603-5629770 - E=7e-89
GGCACTTCCCTGGGGCTTCCTGCTGCGCCCTCCCTGGGGACCCCCTTCTCACCGCAACGTTGCAGCCTCACA
```
